# Damage Tolerance Evaluation of E-PBF-Manufactured Inconel 718 Strut Geometries by Advanced Characterization Techniques

**DOI:** 10.3390/ma13010247

**Published:** 2020-01-06

**Authors:** Daniel Kotzem, Tizian Arold, Thomas Niendorf, Frank Walther

**Affiliations:** 1 Department of Materials Test Engineering (WPT), TU Dortmund University, Baroper Str. 303, D-44227 Dortmund, Germany; frank.walther@tu-dortmund.de; 2 Institute of Materials Engineering–Metallic Materials, University of Kassel, Moenchebergstraße 3, D-34125 Kassel, Germany; arold@uni-kassel.de (T.A.); niendorf@uni-kassel.de (T.N.)

**Keywords:** electron beam powder bed fusion (E-PBF), Inconel 718, damage tolerance, lattice structures, cyclic behavior, defect analysis

## Abstract

By means of electron beam powder bed fusion (E-PBF), highly complex lightweight structures can be manufactured within short process times. Due to the increasing complexity of producible components and the entangled interplay of damage mechanisms, common bulk material properties such as ultimate tensile or fatigue strength are not sufficient to guarantee safe and reliable use in demanding applications. Within this work, the damage tolerance of E-PBF-manufactured Ni-based alloy Inconel 718 (IN 718) strut geometries under uniaxial cyclic loading was investigated supported by several advanced measurement techniques. Based on thermal and electrical measurements, the failure of single struts could reliably be detected, revealing that continuous monitoring is applicable for such complex geometries. Process-induced surface roughness was found to be the main reason for early failure during cyclic loading. Thus, adequate post-processing steps have to be established for complex geometries to significantly improve damage tolerance and, eventually, in-service properties.

## 1. Introduction

Due to urgent demands stemming from sustainability, the efficient use of materials and the reduction of energy spent are recent key challenges in engineering. In this regard, conventional subtractive manufacturing techniques are not able to meet these demands, as complexity of the final components has steadily risen to account for superior lightweight design. Thus, new manufacturing techniques have to be established to fulfill the requirements set. In particular, additive manufacturing (AM) is an indispensable element in regard to the manufacturing of lighter and resource-efficient future components [1], with both aspects being of utmost importance for aerospace and energy applications [2].

Until now, many AM techniques have been available; however, powder bed fusion (PBF) techniques are in focus of many ongoing studies [3,4]. The two most common PBF techniques are laser (L-PBF) and electron beam powder bed fusion (E-PBF). Due to layer-by-layer manufacturing, complex geometries can be built within short process times.

During the E-PBF process, a thin powder layer is spread on the building platform by a rake. Subsequently, the electron beam is used as a high energy source to firstly sinter and afterwards locally melt the powder particles according to computer-aided design (CAD) data [5]. After this, the building platform is lowered and again covered by a new powder layer. In comparison to L-PBF, E-PBF leads to the evolution of different microstructures and material properties [6], amongst others due to differences in beam-powder interactions and process environments, respectively. Metallic alloys, which have already been successfully processed, are Ti-, Fe- and Al-based alloys [7,8,9,10]. Besides PBF, an electron beam is used as an energy source for wire electron beam AM [11,12]. Furthermore, very recent work highlighted the implementation of a plasma electron beam source in AM, as this type of source enables a more robust diameter control and lower vacuum pressure [13].

Focusing on the aerospace sector, the processing of Ni-based alloys, like Inconel 718 (IN718), is of high industrial interest. However, besides all the benefits of AM, new challenges have concurrently arisen. Even though components with a relative density of > 99.5% can be manufactured [6], process-induced features like pronounced surface roughness and other defects like gas porosity or lack of fusion limit the number of potential application fields [14], as these features detrimentally influence mechanical behavior.

Due to the increased complexity of the producible components and the entangled interplay of damage mechanisms, common bulk material properties such as ultimate tensile or fatigue strength are not sufficient to guarantee a safe and reliable use in demanding applications. Therefore, new measurement methods have to be established in order to determine the degree of damage of lattice structures under cyclic loading in situ.

Until now, much research has been carried out focusing on L-PBF manufactured lattice structures [15,16]. Investigations have focused on the influence of process parameters [17], the influence of different build directions [18], and the influence of a supplementary heat treatment on the mechanical performance of lattice structures [19,20].

In regard to the cyclic material behavior of L-PBF-manufactured lattice structures, it has been stated that these properties are mainly affected by the type, size, and relative density of the cell, as well as the mechanical properties of the corresponding bulk material [16,21]. Furthermore, numerical simulations of additively manufactured lattice structures have already been carried out, e.g., by Zargarian et al. [22]. In the aforementioned study the authors concluded that the relationship between cycles to failure and fatigue strength follows a power law; in particular, the influence of specific aspects like relative density and cell topology were highlighted. However, little work regarding the mechanical properties of lattice structures manufactured by E-PBF has been carried out so far [23,24].

As already mentioned, most studies have focused on lattice structures that consist of an relatively high number of linked unit cells. However, as these structures can be highly complex, the determination of damage accumulation, as well as the identification of primary failure mechanisms, is challenging. Thus, single unit cells have to be firstly investigated to allow for the establishment of property-failure relationships. In their investigations, Persenot et al. [14] showed that struts exhibit different mechanical properties as a function of their orientation to build direction. For this reason, the consideration of the behavior of single struts inside a unit cell is mandatory.

To the best of the authors’ knowledge, no profound knowledge on the damage evolution within single struts loaded in parallel, as well as the damage progress in E-PBF manufactured Inconel 718 structures of increased complexity, is available in literature. Within this work, the damage tolerance of single struts is determined under uniaxial cyclic loading by using a combination of load increase (LIT) and constant amplitude (CAT) tests, based on a newly developed specimen geometry. By focusing on the specific strut geometry, two particular aspects are addressed. In addition to the determination of the cyclic material properties of upright manufactured struts, the specific specimen shape is used to simulate the damage behavior of components of more complex shape. For damage tolerance determination, diverse material responses, i.e., the change in electrical resistance and temperature (as well as acoustic emissions), are monitored, and the obtained results are correlated to allow for solid conclusions.

## 2. Materials and Methods

**Figure 1:**
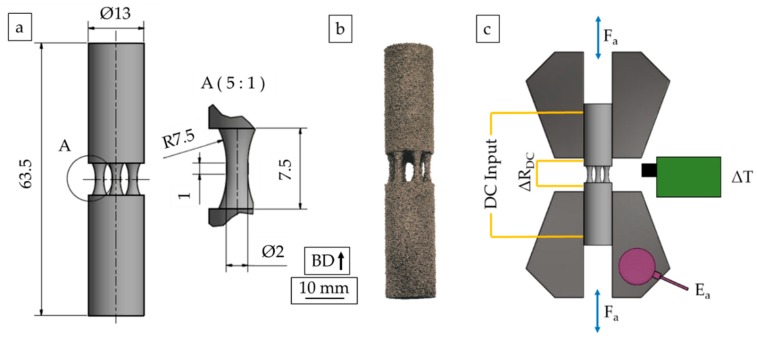
(**a**) CAD geometry, (**b**) final specimen geometry in as-built condition, and (**c**) schematic illustration of the experimental setup used for load increase test (LIT).

The investigations detailed in the following were conducted using E-PBF-processed Inconel 718 alloy (IN718, 2.4668, NiCr19NbMo). Typically, the E-PBF process is divided into four subsequent and repetitive steps: the preheating of the uppermost powder layer for the purpose of presintering, the melting of powder particles with the electron beam as heat source, the lowering of the building platform, and the forming of a new powder layer [25]. Prior to the mentioned steps, the heating of the build chamber up to the desired process start temperature of 1025 °C is done by applying a strongly defocused electron beam that repeatedly scans the build plate. Figure 1a depicts the CAD specimen geometry used in this study. Specimens were manufactured by means of an Arcam A2X machine (Arcam AB, Mölndal, Sweden) applying default process parameters. In the preheat phase, the beam current was set to 48 mA, and the beam speed was set to 15,750 mm/s. The number of repetitions for scanning the current layer during the preheating was between 7 and 20 cycles. In the melt theme, the maximum beam current and the beam speed for hatch melting were set to 18 mA and to 4530 mm/s, respectively. The focus offset was 15 mA, while the speed function was set to 63. For melting of the contours, the beam speed and current were set to 540 mm/s and 8 mA, respectively. Here the focus offset was 3 mA and a speed function of 6 was used. The offset from hatch to contour was 0.3 mm. The parameters detailed were applied to all sections of the bulk material, i.e., the specimen grip sections as well as the struts. The process temperature during the build was constant at 970 °C, and the chosen layer thickness was 75 µm. Based on these parameters, specimens with strut diameters of 2 and 3 mm were manufactured upright. In particular, every specimen consisted of 6 struts. The final as-built specimen geometry can be seen in Figure 1b.

Strut diameters of 2 and 3 mm exceed the typically considered strut diameters being realized by AM. However, it has to be emphasized that, especially in the case of the E-PBF process (based on the use of the Arcam standard parameters for Inconel 718), the smallest geometrical feature size manufacturable is about 1.2 mm. Every geometry that is intended to be smaller than this size appears in the processed part as a feature with a size of about 1.2 mm. To assure that the size and the sphericity of the cross section of the processed struts were close to the cross section of the struts of the initial CAD file, strut diameters of 2 and 3 mm were chosen for the specimens used in this study. Changes in local microstructure indicated that the thermal history was fundamentally different in these structures, a fact that is characteristic for any kind of strut, as detailed in the literature for L-PBF processed specimens [26].

For the detection of process-induced features like surface topography and porosity, specimens with a strut diameter of 2 mm were probed by computed tomography (µ-CT). The system used was an XT H 160 (Nikon Metrology, Tokyo, Japan) with a maximum acceleration voltage of 160 kV. The chosen scanning parameters are listed in Table 1. The scanned volumes fully covered all critical specimen regions. The transition area to the bulk grip sections was not investigated. For the µ-CT analysis of the reconstructed volumes, the software VG Studio Max 2.2 (Volume Graphics, Heidelberg, Germany) was used.

In order to determine the fatigue properties of the strut specimens, load increase (LIT) and constant amplitude (CAT) tests were carried out in a combined fashion. A servohydraulic system Schenck PC63M (Instron^®^, Norwood, MA, USA), equipped with a 45 kN load cell, was used. All experiments were carried out at a test frequency of f = 10 Hz in fully reversed tension-compression (R = −1) loading. In case of the LIT, the initial load started at σ_a_ = 5 MPa and was increased stepwise by 5 MPa after every 10^4^ cycles. Additionally, material responses were recorded by application-specific instrumentation. In detail, an infrared camera (Micro-Epsilon, Ortenburg, Germany) was used to determine deformation-induced temperature changes during the experiment. To allow for precise measurements, specimens were colored with a black coating. Furthermore, a direct current potential drop (DCPD) measurement system (Ametek^®^, Berwyn, PA, USA) was used to capture changes in electrical resistance. Finally, an Optimizer4D sensor (Qass, Wetter, Germany) was chosen for the detection of acoustic emissions. Taking into account the ambient noise of the servohydraulic testing system mentioned above a threshold value was defined for the acoustic emissions. Acoustic emissions that exceeded this threshold were subsequently accumulated. Thus, the maximum sum of the acoustic emissions represented final failure of the specimen. Based on this value, acoustic signals were normalized. In the following sections, the term normalized accumulated acoustic emission (E_a_), analogous to [27], is used. A schematic illustration of the experimental setup is presented in Figure 1c.

As a result of initial screening, stress amplitudes of σ_a_ = 105, 95, and 85 MPa were selected for the CAT. At least two specimens of each batch were tested at the aforementioned stress amplitudes. The stress amplitude was calculated based on the actual diameter of the six struts, and this diameter was determined prior to mechanical characterization by a caliper. After fatigue testing, fractographic analysis was carried out using a scanning electron microscope (SEM) Mira 3 XMU (TESCAN, Brno, Czech Republic).

In addition, microstructural investigations were carried out on a specimen after mechanical testing. The gauge length of the fatigue specimen, consisting of six struts, was cut out by electrical discharge machining (EDM). The cut was accomplished directly above and below the struts on a plane perpendicular to the longitudinal axis of the specimen. A second cut was carried out alongside the build direction (BD) on a plane that was defined by the longitudinal axis of two adjacent struts. For the characterization of the microstructure, a Cam Scan MV2300 SEM (TESCAN, Brno, Czech Republic) equipped with electron backscatter diffraction (EBSD), secondary electron (SE) and backscatter electron (BSE) detectors was used.

Since the hardness values of IN718 are strongly affected by the volume fraction of the hardening phases γ′ and γ″, as well as detrimental phases such as the Laves- and δ phases, hardness measurements were conducted to reveal the homogeneity of distribution of the hardening phases. Most importantly, the cross section transition areas, i.e., the areas from the specimen grip section to the struts, had to be characterized. Measurements were done for one specimen with struts of 2 mm diameter in a plane parallel to the BD in the upper and lower transition areas of the specimen. For the measurements, a Leco V100 hardness testing machine (Leco GmbH, Emsdetten, Germany) was used. The testing force was set to 9.81 N (HV 1). For the hardness measurements, an area of 600 × 900 µm was probed based on a matrix that included 3 × 4 measurement points. All measurements were carried out in the center part of the strut at about 600 µm distance to the strut surface.

## 3. Results

The results of the hardness measurements that were carried out on a strut with a 2 mm diameter are listed in Table 2. Each specimen volume probed revealed a direct aged condition, e.g., the hardness values found were similar to the hardness of conventionally manufactured IN718 after aging [28]. The hardness values in the different specimen areas did not significantly differ from each other, and, thus, despite the pronounced changes in geometry in the characterized specimen areas, homogeneous local mechanical properties were found.

**Figure 2:**
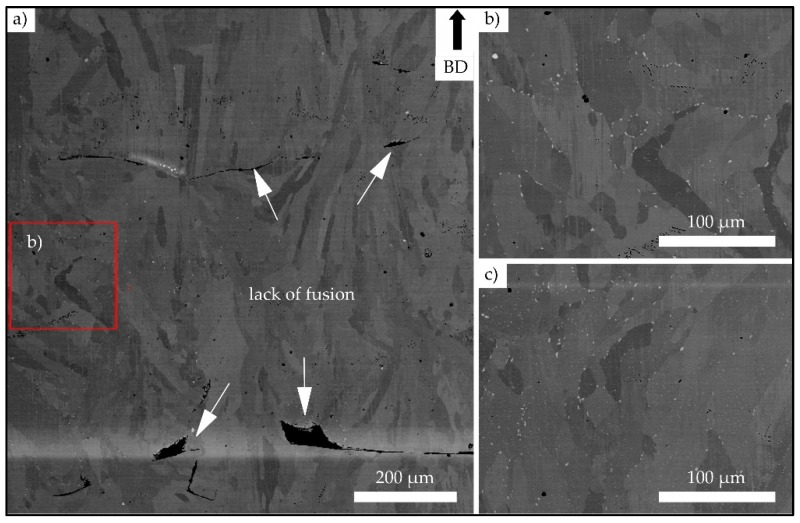
Backscatter electron (BSE) images of the lower half of one strut with a 3 mm diameter after initial testing. (**a**) Overview detailing the microstructure within an area from the left strut surface to the center line of the strut; (**b**) the area marked red in (**a**) at higher magnification; and (**c**) a specimen region below the area visible in (**a**) for reference.

**Figure 3:**
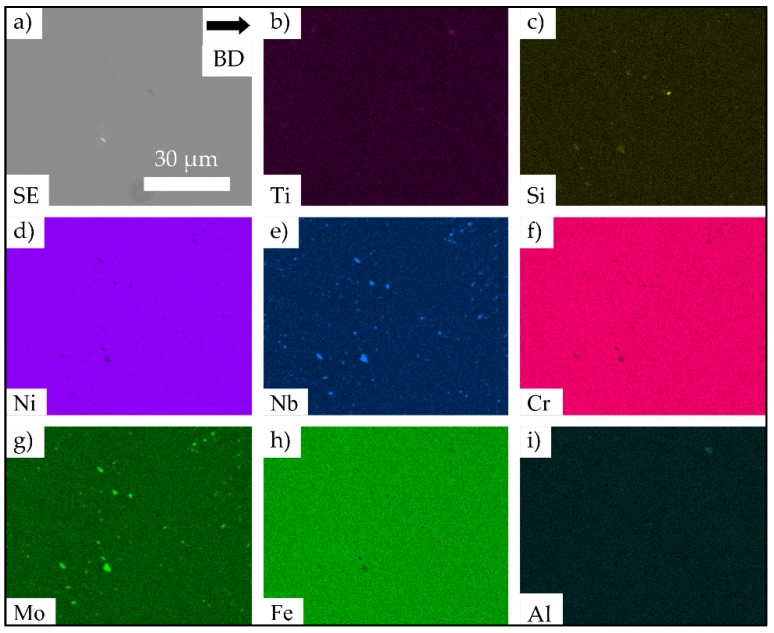
EDS mappings of an area close to the strut surface in direct vicinity of the final crack that was found within the lower half of a strut with a 3 mm diameter after initial testing. (**a**) Reference SE image. (**b**–**i**) EDS maps for the elements Ti, Si, Ni, Nb, Cr, Mo, Fe and Al.

Figure 2 shows the BSE images of the lower half of one strut after initial testing. The areas depicted in Figure 2a–c were sited in direct vicinity of the fracture surface of the strut. Large lack of fusion defects can be seen in Figure 2a. The white features shown in Figure 2b,c were further characterized by means of energy-dispersive X-ray spectroscopy (EDS). The EDS mappings depicted in Figure 3 reveal a localized increase of Nb and Mo and a decrease of Cr, Fe and Ni at these points. This kind of localized chemical segregation can be interpreted as a strong evidence for the presence of the brittle Laves phase. However, a clear separation of carbides and Laves phase by EDS is hardly possible, and, thus, the presence of carbides cannot be excluded. Near to the surfaces, the formation of carbides and/or the Laves phase was more pronounced in comparison to the center line of the strut, where almost no white features could be seen. The precipitations close to the specimen surface formed either on grain boundaries (shown in Figure 2b) or dispersedly throughout the grains, as is depicted in Figure 2c.

**Figure 4:**
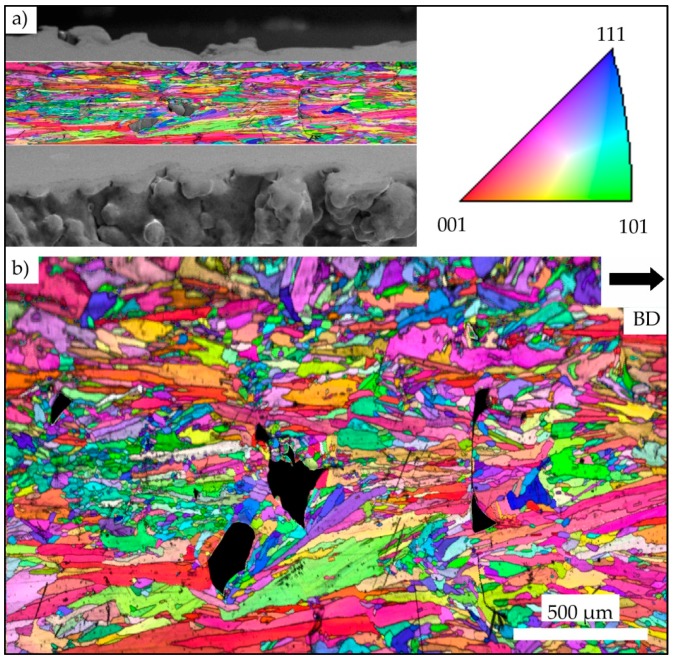
Inverse pole figure (IPF) map of a strut with 3 mm diameter highlighting a plane parallel to the build direction (BD) with grain orientations plotted for the BD. (**a**) Mapped area with reference to the overall strut dimensions; (**b**) probed volume depicted in normal view for further analysis. All defects already seen in (**a**) are colored black in (**b**). Color coding is according to the standard triangle shown in the upper right.

Figure 4a highlights the investigated area of a strut with superimposed EBSD information. Figure 4b provides for a normal view on the area investigated for clarity, showing an inverse pole figure (IPF) map with grain orientations plotted with respect to BD. The investigated area represents a part of the strut that ranges from the fracture surface on the right to the transition point from strut to the bulk material of the specimen grip section on the left. For clarity, all defects are colored black. Figure 4b reveals a multimodal microstructure that is characterized by fine equiaxed grains with a size of only several microns next to elongated grains with dimensions ranging from few microns up to about one millimeter (with respect to the grain long axis). Within the probed area of the strut, the IPF map reveals no significant texture and anisotropy, respectively. 

**Figure 5:**
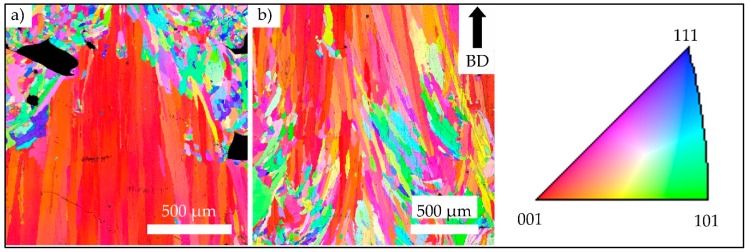
IPF maps plotted for BD depicting the transition areas from the lower specimen grip section to the strut with a 3 mm diameter in (**a**) and the strut to the upper specimen grip section in (**b**). Defects are colored black; color coding is according to the standard triangle shown on the right.

Figure 5a shows the transition point from the lower specimen grip section, i.e., bulk material, to the strut. The bulk material is characterized by a strong < 001 > texture parallel to BD. Upon transition into the strut, the microstructure significantly changes. Grain morphology is equiaxed and texture intensity is weak, respectively, and lack of fusion defects concomitantly occur (see Figure 5a). Within the transition area from the strut to the upper bulk part of the specimen, as seen in Figure 5b, the microstructural appearance changes the opposite way, i.e., from hardly textured and equiaxed microstructure to strongly textured and columnar grains.

### 3.1. µ-CT Analysis

**Figure 6:**
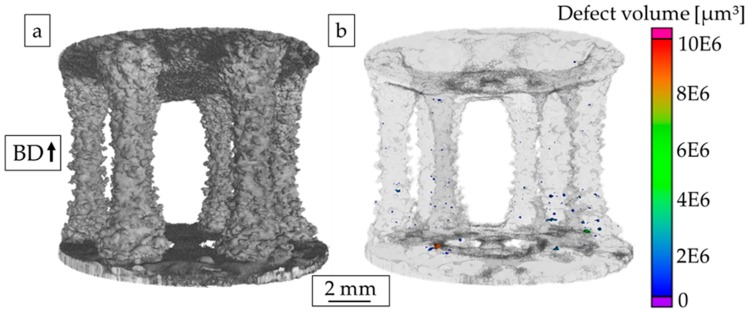
(**a**) Reconstructed µ-CT-volume and (**b**) pore distribution within the struts of one specimen (strut diameter of 2 mm).

A reconstructed µ-CT volume of an exemplary specimen with a strut diameter of 2 mm is shown in Figure 6a. As can be seen, the specimen surfaces are characterized by an irregular topography. In particular, partially melted powder particles can be detected, and “plate-pile” like stacking defects are present. Further on, the process-induced pores and their locations inside the corresponding strut specimen are presented in Figure 6b. The resolution limit of the µ-CT at the given working point was 12 µm.

**Figure 7:**
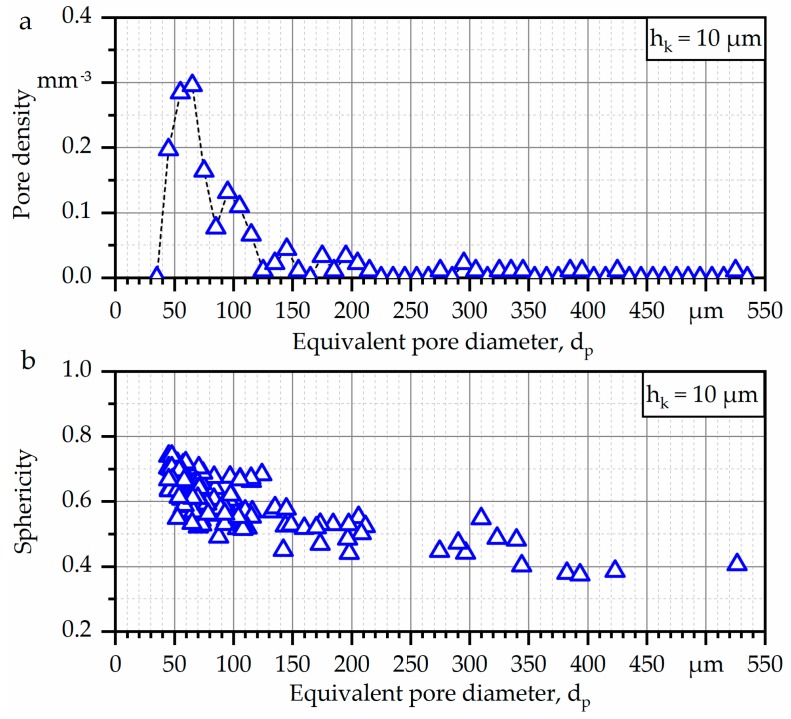
Characteristics of defects being present within the specimen (strut diameter of 2 mm): (**a**) pore density and (**b**) sphericity.

Furthermore, a quantitative analysis of the prevailing defects was carried out, as shown in Figure 7. Figure 7a highlights the pore density as a function of the equivalent pore diameter d_p_. The equivalent pore diameter is divided into classes (d_p_^−5^, d_p_^+5^) in order to assist analysis. The total analyzed volume for the investigated specimen was around 91 mm^3^, and the relative density was found to be > 99.9%. With regard to the pore density, the overall number of pores was found to be low, and only one pore exhibited an equivalent pore diameter > 500 µm. Additionally, it can be stated that both spherical and elongated pores could be found (Figure 7b). The biggest pore, which also had the lowest sphericity, was located close to the transition to the lower specimen grip section. As stated before, the grip section itself was not part of the investigations.

### 3.2. Geometrical Deviations

The effective diameters of at least three specimens for each type of strut geometry were measured with a caliper, and the results are presented in Table 3. As can be seen, both types of specimens were characterized by an undersized effective diameter. In particular, the effective diameter for the specimens with a nominal diameter of 2 mm (according to CAD data) was found to be 1.75 mm. In comparison, the specimens with a nominal strut diameter of 3 mm had an effective diameter of 2.82 mm. The standard deviations for both specimens were found to be low, revealing that every specimen suffered by the undersized diameter in the same way. The variations in diameter between single struts were found to be low. However, the determined effective diameters led to different deviations when compared to CAD data for both types of struts. In detail, the deviations to the CAD data were found to be 12.7% for the smaller 2 mm struts and 6.0% for the bigger 3 mm struts.

### 3.3. Mechanical Properties

**Figure 8:**
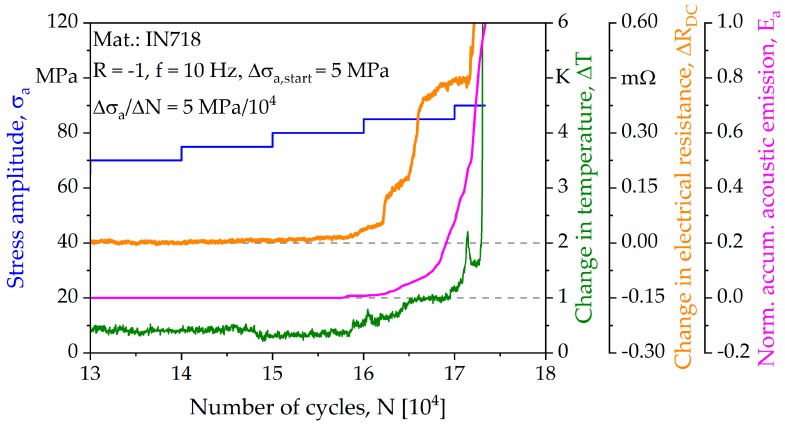
Material responses recorded to be used for the analysis of deformation and damage progress in the load increase test.

The load increase test (LIT) was carried out for a specimen with a strut diameter of 2 mm, and the corresponding results, highlighting different material responses, are plotted in Figure 8. In the diagram, stress amplitudes (σ_a_) are colored in blue, changes in temperature (ΔT) in green, changes in electrical resistance (ΔR_DC_) in orange, and normalized accumulated acoustic emissions (E_a_) in magenta. As can be seen, a maximum stress amplitude of σ_a_ = 90 MPa and a total number of cycles N_f_ = 173,323 were achieved. Referring to the material responses highlighted, a first reaction in the form of a linear increase could be detected through all measurement techniques at a stress amplitude of σ_a_ = 80 MPa. Before, all measurement techniques were characterized by an almost horizontal, i.e., constant, course. Upon an initial linear increase of signals, the trendlines are characterized by an exponential increase, finally ending in failure of the specimen.

**Figure 9:**
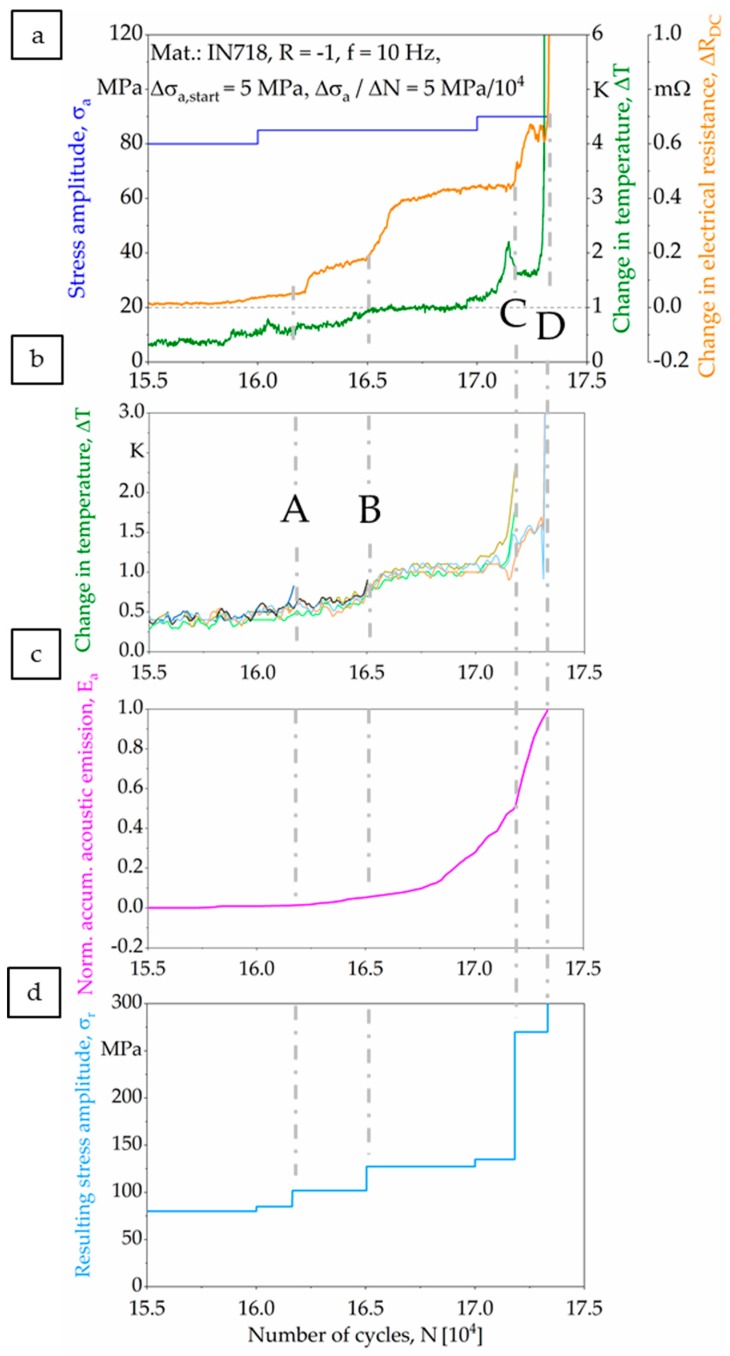
Detailed presentation of material responses that were recorded during the load increase test before failure: (**a**) change in temperature and electrical resistance, (**b**) specific change in temperature for single struts, (**c**) normalized accumulated acoustic emissions, and (**d**) evolution of nominal stress for remaining struts.

Based on the aforementioned results, the interval between the first material response and final fracture was analyzed in detail and is plotted in Figure 9. Figure 9a highlights the interrelations between the actual load level and the evolution of temperature and electrical resistance. For a better interpretability of results, the temperature changes of single struts were separated and are plotted in Figure 9b. The normalized accumulated acoustic emissions are depicted in Figure 9c. Furthermore, the boundaries of the characteristic intervals of the LIT are labelled as A–D. Each interval can be related to the failure of a single strut. Based on an in-depth analysis of data, the resulting stresses were calculated for the remaining struts after each incident (Figure 9d).

**Figure 10:**
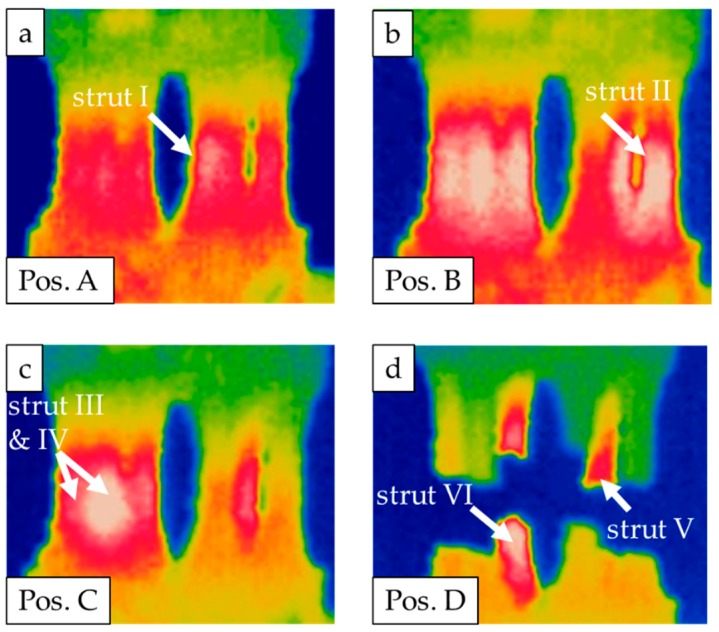
Thermography images of single strut failure: (**a**) strut I, (**b**) strut II, (**c**) struts III and IV and (**d**) struts V and VI.

Additionally, the corresponding thermography images of the specimen considered (strut diameter of 2 mm) are presented in Figure 10a–d, where the failure of a strut is marked with white arrows in each single image. Obviously, a first material reaction could be detected by all techniques employed. As can be deduced from direct comparison, all measurement techniques captured the material reaction almost simultaneously.

At point A, the change in material response in terms of the signals recorded can be definitely linked to the failure of the first strut based on analysis of the thermography image (see Figure 10a). Concurrently, this failure event was detected through a change in temperature and in electrical resistance (see Figure 9a), as both measurement techniques showed a significant increase in absolute values. After this, the resulting nominal stress amplitude increased for the remaining struts to 102 MPa. The second strut failed in point B (see Figure 10b). At this point, in the acoustic emissions signal only a slight increase could be detected, whereas the change in electrical resistance was more significant. Afterwards, the resulting nominal stress amplitude increased to 127.5 MPa. At point C, the simultaneous failure of two struts was observed (see Figure 10c), and significant changes in electrical resistance and acoustic emissions could be resolved. The simultaneous failure of two struts led to an increase of the resulting nominal stress amplitude to 270 MPa. On the basis of the normalized accumulated acoustic emissions, a slope increase could be detected within the interval ranging from B to C. This increase could not be attributed to failure of a strut. At point D, the two remaining struts failed (see Figure 10d). The struts did not fail simultaneously; rather, they failed with short delay. As can be seen in Figure 9, all measurement techniques could reliably detect the final failure.

**Figure 11:**
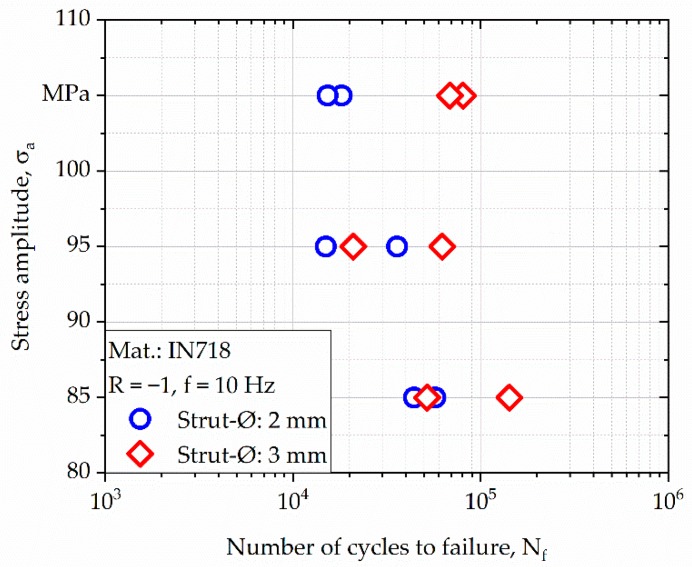
Results of constant amplitude tests (CATs) depicted in form of a typical Woehler-type S–N plot.

Based on the results from the LIT, three stress amplitudes (85, 95, and 105 MPa) were chosen for the constant amplitude tests (CATs). Results from the CATs are plotted in Figure 11. As can be seen, deviations in the number of cycles to failure (N_f_) were present in case of all tested stress amplitudes. However, specimens with a strut diameter of 3 mm showed better overall cyclic properties, especially at higher stress amplitudes.

### 3.4. Fractography

**Figure 12:**
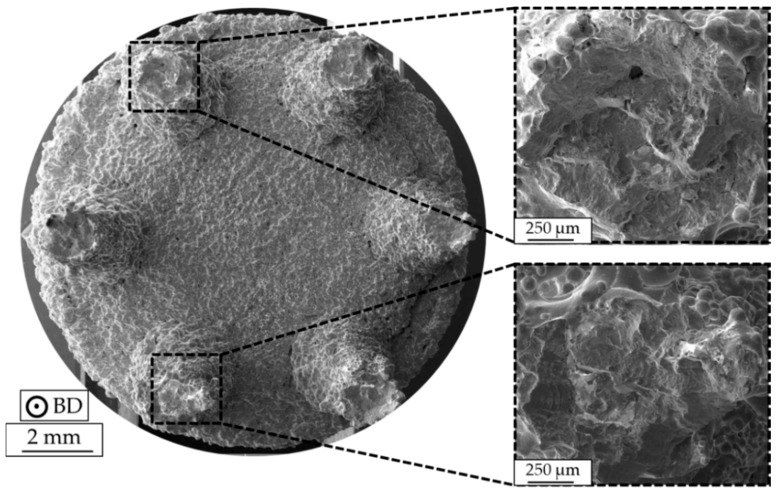
Fracture surface of an as-built specimen with a strut diameter of 2 mm, tested at a stress amplitude of σ_a_ = 105 MPa (N_f_ = 18,174 cycles).

As can be seen in Figure 11, pronounced deviations with respect to fatigue lives prevailed on individual stress levels for the as-built specimens with different strut diameters, especially at higher stress amplitudes. To further provide data on the respective characteristics of single struts, specimens were further investigated by fractographic analysis. An exemplary fracture surface of a specimen with a strut diameter of 2 mm, tested at 105 MPa (N_f_ = 18,174 cycles), is depicted in Figure 12. Furthermore, two corresponding fracture surfaces of single struts are presented at a higher magnification. As can be seen, fracture surfaces show significant deviations in relation to an ideal circular cross-section, revealing that the actual diameter differed not only in terms of cross section in comparison to the initial CAD data. However, fracture surfaces do not show traces of lack of fusion defects, and only gas pores seem to be present (as already indicated by the results obtained by the µ-CT analysis) within the strut volumes that are characterized by minimum diameter, i.e., at about half the height of the struts.

**Figure 13:**
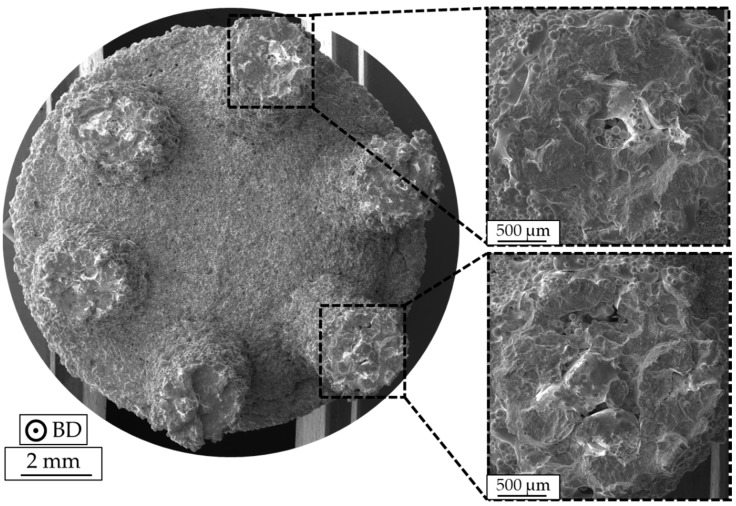
Fracture surface of an as-built specimen with a strut diameter of 3 mm, tested at a stress amplitude of σ_a_ = 105 MPa (N_f_ = 80,737 cycles).

In Figure 13, the fracture surface of a specimen with a strut diameter of 3 mm, which also was tested at 105 MPa (N_f_ = 80,737 cycles), is presented. Two fracture surfaces of single struts are shown at higher magnification. In contrast to the results shown before, significant differences on the fracture surface of single struts are visible. As can be seen, almost all struts suffered lack of fusion defects. As can be seen in Figure 13, the struts are characterized by entrapped, partially melted powder particles. These defects are mainly located in the center of the struts. At the same time, the shape of the cross-sections almost equals an ideal circular shape. For both specimens, single crack initiation events could not be detected. Moreover, multiple crack initiation sites are present. Nevertheless, crack initiation is dominantly seen at the specimen surface, revealing that surface roughness is the main reason for the early failure of as-built components despite internal defects being present.

## 4. Discussion

### 4.1. Microstructure

The general appearance of the IPF maps plotted for BD, which were obtained from the specimen grip sections (shown in Figure 4a,b), is in excellent agreement with the well-known microstructure established by E-PBF, e.g., columnar grains elongated in BD featuring a strong <001> texture [29,30,31]. This microstructure can be ascribed to the standard cross-snake scanning strategy, the melt pool shape, the resulting steep temperature gradient being primarily aligned to BD [32,33] and, eventually, to the overall thermal history being induced by the process and its parameters. The IPF map that was obtained from a section of a representative strut plotted in Figure 4b does, however, show a multimodal microstructure that is composed of epitaxial grains and relatively small equiaxed grains. The equiaxed grains are predominantly characterized by sizes ranging from 20 to 80 µm. Besides the relation of thermal gradient and solidification velocity, further superimposed factors are known to affect microstructure evolution in terms of epitaxial or equiaxed grain growth. Ding et al. [34] attributed the presence of equiaxed grains to the presence of volumetric defects, where the defect surfaces act as heterogeneous nucleation sides. It is expected that this mechanism has a minor impact in case of the specimens that are shown in the present study. Still, defects being present could have led to the microstructure evolution seen in parts of the specimen shown in Figure 4a, where fine equiaxed grains grew alongside the BD in direct vicinity of the lack of fusion volumetric defects colored in black. In case of a relatively high porosity level inside single struts, e.g., as highlighted in Figure 4b, Figure 6b and Figure 13, epitaxial growth in a stable fashion is somehow hindered within these struts. The second reason for weak texture and equiaxed grain growth is expected to be the strongly affected thermal conditions in the layers containing the struts. Here, it is very important to note that the surrounding powder shows very low thermal conductivity in comparison to the bulk material. Furthermore, the absolute energy input per layer is significantly lower, as only the strut volumes are irradiated. Effects stemming from these factors are expected to be even more pronounced in the cross section transition areas. Here, the lateral width of the molten volume changes rapidly within only a few layers. Though scanning speed and beam power may vary in a certain range (due to the auto functions being generally activated in the standard process theme to maintain the melt pool geometry), it can be expected that because of the strong cross section reduction from specimen bulk material to the struts, thermal fields within the struts are significantly different as compared to the bulk specimen areas. Thus, melt pool size, thermal flux and solidification conditions are eventually changed. In case of the specimens that are considered in the present work, this is expected to lead to varying local temperatures in the strut and, hence, eventually to the melt pool being significantly different in terms of general shape. Again, in the transition areas, fundamentally different boundary conditions prevail. It is well known that the melt pool shape significantly affects microstructural appearance upon solidification [35]. Furthermore, return times and, thus, the temperature of previously melted regions, are differently affected. All factors mentioned and their complex interplay are thought to lead to pronounced differences in solidification conditions. For in-depth analysis, modeling approaches have been proven to be highly beneficial for the rationalization of experimental findings [36,37]. However, modeling of solidification is clearly out of the scope of the present work.

For the E-PBF-processed IN718, lack of fusion defects close to the surface have already been highlighted in literature. Balachandramurthi et al. [38] attributed this kind of defects to inadequate process parameters resulting in a too large distance from the hatch pattern to the contour melt track and, additionally, to embedded aluminum oxides. In the aforementioned study, oxides were shown to originate from the helium process atmosphere of the E-PBF process, which is not pure enough to prevent the oxidation of particles during spatter injection. As in the bulk specimen sections such defects are significantly lower in terms of number fraction and, as process parameters applied in previous studies have been proven to lead to material of good structural integrity [39], both factors are expected to be of minor importance only. As is shown by the µ-CT results presented in Figure 6, the distribution of defects is not homogeneous in the struts. The highest density of defects is found in the lower transition region, where cross sections and, thus, thermal fields changed rapidly. Based on these findings, it is assumed that the significant changes in thermal conditions, as discussed in detail above, are responsible for the locally different evolution of defect density. However, how far the cross section dimensions of one single strut in detail contribute to the distribution and number of defects is not fully understood yet and remains part of ongoing work. The same holds true for the locally different evolution of precipitates, i.e., Laves phase and/or carbides, as shown in Figure 2 and Figure 3. The Laves phase (Ni, Cr, Fe)_2_(Nb, Mo, Ti) is a hexagonal, brittle phase that forms in IN718 due to the high segregation rate of Nb in the interdendritic regions during the solidification process [40]. In the SEM BSE micrograph a high local niobium content can be seen as white features (shown in Figure 2b,c) due to the higher element contrast (Z-contrast). These results are further promoted by the EDS results, as shown in Figure 3. The BSE analysis, shown in Figure 2a, reveals only a minor fraction of the Laves phase in zones near to the surfaces of the struts. The areas close to the center line of one strut are virtually free of Laves phase. The common array-like segregations pattern of the Laves phase, as shown for example by Deng et al. [41], are not visible. Instead, the Laves phase only formed along grain boundaries (Figure 2b) or is dispersedly distributed within a grain (Figure 2c). Xiao et al. [42] ascribed such a disperse precipitation pattern to the fast cooling rate being characteristic for the L-PBF process. Hardness measurements revealed a relatively homogeneous hardness level in the struts, as is expected for a direct aged condition similar in hardness to wrought and aged IN718 [28]. It is important to note that both the Laves phase and the hardening phases γ′ and γ″ consume niobium for their formation. The dissolution temperature of the Laves phase is between 982 and 1093 °C [43], while γ′ and γ″ precipitates form during the cooling phase at about 900 °C [44]. As already detailed above, it can be concluded that during the build, the temperatures within the strut deviated significantly from the bulk, eventually leading to the different appearance of the microstructure within the struts. Still, homogeneity in terms of hardness is sufficient to prevent localized weakening of the areas in the transition area, as is discussed in the remainder of the present work.

### 4.2. µ-CT Analysis

Due to the complex shape, the material employed, and overall dimensions of the specimen type investigated, the resolution limit of µ-CT had to be decreased in order to penetrate the complete geometry. Therefore, no defects below a resolution limit of 12 µm could be detected in the present investigations. In regard to the µ-CT results (Figure 6a), it could be revealed that E-PBF-manufactured IN718 as-built components mainly suffers from high process-induced surface roughness, which, in particular, is known to be higher than the surface roughness of L-PBF-manufactured components [45]. In detail, surface imperfections, e.g., “plate-pile”-like stacking defects and partially melted powder particles sticking on the surface, can be seen. Such defects were already earlier reported, e.g., in [24,46,47]. Heterogeneous surface topography has been identified to be caused by varying melt pool shapes during the manufacturing process and the scanning strategy applied [46]. In the present work, the overall internal porosity is found to be low (Figure 6b), and only a slightly increased number of pores is found for some critical regions in the investigated specimens (Figure 6 and Figure 7), implying that virtually fully-dense specimens were obtained. Thus, E-PBF-manufactured IN718 components featuring high complexity and locally small features can be obtained. Nevertheless, process-induced defects are unavoidable [48]. These defects can typically be divided into gas- or process-induced porosity. The formation of process-induced internal pores is mostly attributed to non-adequate process parameters [49]. Out of these populations, relatively large defects with a flat and elongated shape that are oriented perpendicular to the loading direction were found to be critical under cyclic loading [50]. In the present work, an increased fraction of pores is found especially in the transition regions. These regions are proven not to be critical in terms of fatigue failure. Fatigue crack initiation and final fracture occur in the specimen section that is characterized by minimal cross section and, thus, not in the region being characterized by highest pore density. Furthermore, crack initiation is induced at the specimen surface. Thus, even if process parameters applied for manufacturing are not optimized to allow for defect-free production in transition areas, the presented results are representative for the failure of the struts, e.g., the E-PBF-manufactured parts being characterized by relatively small dimensions.

### 4.3. Mechanical Properties

Following design and adequate material selection, the determination of fatigue-related material heterogeneities is of high importance in order to enable a reliable operation [51]. In the case of cyclic loading, a combination of multi and single step tests can be used to estimate fatigue life in a time- and cost-efficient way [51]. For final fatigue life calculation, several physical measurement methods are available to obtain further information about the actual material state during cyclic loading.

Typically, material changes are captured by means of the plastic strain amplitude; however, Piotrowski et al. [52] demonstrated that temperature and resistance measurement techniques can be used supplementary to gain additional details about the cyclic behavior of a material. Temperature changes during fatigue tests can be straightforwardly attributed to dissipated energy resulting from plastic deformation [52,53]. However, the change of electrical resistance is a more complex measure. Besides being affected by geometry induced changes of component cross sections, the electrical resistance depends on the specific electrical resistance, which is known to be influenced by the actual defect density and the effective cross section being affected by crack initiation and propagation [52,53]. Mostly, it is assumed that the determined physical values can be directly correlated to the actual fatigue state and, thus, the damage progress. However, a complex interaction of signals has been revealed [23] such that a simultaneous application of several measuring techniques should be considered. In the literature [53,54], the combination of these techniques has been successfully applied for the detailed evaluation of the fatigue behavior of various steels and lightweight alloys.

Within this work, a combination of load increase (LIT) and constant amplitude (CAT) tests is applied in order to capture the damage behavior and corresponding fatigue performance of the strut specimens processed by E-PBF, in line with procedures already adapted in previous studies [51]. Application-specific measurement techniques are used to record the material response during the LIT (Figure 1c). Measurement techniques are further supplemented by an additional sensor in order to record acoustic emissions (AE). Huang et al. [55] already reported that the cumulative AE can be used to characterize crack initiation and propagation events during cyclic loading. It has been further stated in literature that an increased level of AE signals can be attributed to crack-tip plastic deformation and transgranular cleavage [55,56].

Based on the recorded material responses, it is possible to fully analyze the damage development and accumulation within the studied complex geometry (Figure 8). In order to evaluate the damage tolerance of the used specimens in-depth, the period between initial changes of the signals monitored and final failure is further analyzed (Figure 9). In particular, temperature development of single struts is studied and evaluated based on the thermography images (Figure 10). It could be revealed that local deformation and failure of single struts always leads to a pronounced local increase in temperature, which can be attributed to the dissipated energy caused by plastic deformation. Based on the exact determination of single strut failure, the resulting stress amplitudes of the remaining struts can be determined. Eventually, a local accumulation of stresses is visible, ending up in specimen failure. However, final failure does not occur immediately after the first failure of a single strut, i.e., on the load level of the first failure event. Instead, the load levels in the LIT can be further increased, and the remaining struts are able to robustly accommodate many more cycles before final failure. The maximum resulting stress amplitude in the tests is found to be 270 MPa. Besides the increase in temperature, the failure of single struts also leads to a characteristic change in electrical resistance (Figure 9), which can be attributed to the decrease of (remaining) effective cross section. The change in electrical resistance is pronounced for the investigated specimen, implying that resistance and thermometric measurement methods can be applied for condition monitoring in order to detect early damage initiation. However, further investigations are needed in order to prove how a further increased complexity of the component influences the implementation of these methods, as well as the absolute values of the output signals.

The failure of single struts could not be robustly determined by means of the cumulative AE in the present work. Nevertheless, characteristic stages during the fatigue test can be highlighted (Figure 9c), in line with the results of Huang et al. [55]. According to their work, three characteristic stages can be detected during a fatigue test, whereby the first stage corresponds to the cycles before the cyclic stress–strain curve stabilizes. The second stage, which is also known as the crack-incubation stage, is less informative, and only the third stage is characterized by AE signals significant in terms of absolute value. These signals can be related to crack growth and propagation. In case of the present work, similar stages are visible for the investigated strut specimen, whereby the transition between the second and third stage was found to be most significant, corresponding to macro-crack initiation [55]. In regard to condition monitoring, the detection of this characteristic point can be used to detect fatigue damage at very early stages, as was already proposed by Mazal et al. [57].

Results from the LIT were used to select single stress levels for the following CATs (Figure 11). The fatigue characterization of the E-PBF-processed Inconel 718 bulk material was the subject of previous work. The results detailing the fatigue behavior of bulk material as basis for the comparison with the strut specimens can be found in [58]. As depicted in Figure 11, specimens with a strut diameter of 3 mm show better cyclic properties than specimens with a smaller strut diameter. In order to address this point, geometrical deviations of single struts were supplementary determined. As shown in Table 3, both type of specimens suffer from an undersized diameter, which was already reported by Suard et al. [59]. It could be revealed that geometrical deviations are less pronounced with increasing strut diameter, implicating that the supporting cross section is higher, which might explain the superior fatigue life (Figure 11) of the specimens with the bigger strut diameter. Similar findings were reported by Dong et al. [60] and Fotovvati et al. [61], both assuming that size-dependent shrinkage is the main reason for dimensional heterogeneities in small-scale struts. Furthermore, the size effect [62] has to be considered, assuming that a larger volume has a higher probability of forming fatigue-initiating defects. Finally, the different surface-to-bulk volume ratios have to be considered at this point. Upon crack initiation, crack advance is expected to follow similar crack growth rates that are independent of the strut diameter. Thus, crack initiation at the most detrimental surface flaw determines fatigue life [58]. Eventually, the influence of geometrical deviations seems to be predominant in the present work. A high variance in fatigue life is present, even for specimens with a similar strut diameter. Based on the fractographic analysis (Figure 12 and Figure 13), it can be identified that multiple crack initiation sites are present. In their investigations, Kahlin et al. [63] showed that irregular as-built surface topography can lead to local stress concentrations, which decrease fatigue strength and cause a high scatter in fatigue life.

As mentioned above, complex geometries show a different damage behavior when compared to bulk material, and numerous features affect final fatigue life. Thus, the determination of a general fatigue limit for E-PBF IN718 in an as-built state, especially for complex geometries, is still a challenge, and further investigations are needed. However, based on this study, it could be demonstrated that condition monitoring is applicable for complex geometries in order to enable an application in safety-relevant components. In particular, thermometric and electric measurement techniques are shown to be highly promising, as single strut failure could reliably be detected. Nevertheless, a combination of several measurement techniques enables a better insight in order to fully understand damage behavior. Future investigations will focus on more complex geometries, e.g., lattice structures. Based on the results shown in the present work, however, it can already be deduced how to transfer the measurement approach established, to ensure the reliable detection of the material responses with significantly increased number of struts.

## 5. Conclusions

In the present study, the damage tolerance of E-PBF-manufactured IN718 under uniaxial cyclic loading was investigated by means of thermal, electrical and acoustic measurement techniques. A newly designed strut specimen was used. To evaluate the mechanical behavior, a combination of load increase and constant amplitude tests was employed, and the corresponding material responses were recorded.

It is shown that a reduction in cross-section size leads to a change in microstructure from highly textured, columnar grains to a multimodal microstructure featuring equiaxed and columnar grains. The (for the E-PBF process well-known) characteristic anisotropic microstructure of IN718 is restored after the second cross section transition towards bulk material. Despite the change in microstructure caused by the cross-section reduction, the hardness over the build height remains constant and, furthermore, is similar to conventionally processed, i.e., wrought and aged, IN718 alloys.

These results demonstrate that all applied measurement techniques can generally detect primary damage. In particular, the failure of single struts can be reliably detected by means of thermometric and electric measurement techniques, clearly revealing that continuous monitoring is applicable for complex geometries in order to follow the progress of damage.

As revealed by the employed experimental approach, microstructure changes and increased defect density in the struts and transition regions do not have a dominating detrimental effect on the overall performance of the struts. It is expected that failure is primarily induced by the rough surface and more specifically, by the most severe surface flaw of the struts within the area, which is characterized by smallest diameter. Surface finishing, hot isostatic pressing, and heat treatments will be applied in future works in order to support this assumption. These further investigations, as well as an increased number of tested specimens, are needed in order to statistically validate the presented findings.

Furthermore, in light of the increased complexity of modern lightweight components, e.g., in case of lattice structures, the influence of process parameters on the process-induced surface and inner defects, as well as corresponding mechanical properties, has to be further investigated. The irregular surface topography, which will be even more pronounced when struts are oriented differently, is the main reason for early failure during cyclic loading. Additionally, adequate post-processing steps have to be established for complex geometries in order to reduce surface roughness and the presence of notch-like defects, which currently lead to local stress concentrations and eventually rapid crack initiation.

## Figures and Tables

**Table 1:** Scanning parameters for computed tomography (µ-CT) investigations.

Material	Beam Energy	Beam Current	Power	Effective Pixel Size	Exposure
IN718	160 kV	78 µA	12.48 W	12 µm	354 ms/2.82 fps

**Table 2:** Vickers hardness distribution over build height for a strut with a 2 mm diameter.

Specimen Area	Build Height [mm]	Hardness [HV 1]	Standard Deviation [HV 1]
Upper specimen grip section	37	416	5
Upper transition area	35	430	17
Lower transition area	32	422	11
Lower specimen grip section	26	422	9

**Table 3:** Geometrical deviations being characteristic for the strut geometries.

CAD Strut Diameter	Effective Strut Diameter	Deviation
2 mm	1.75 ± 0.01 mm	12.7%
3 mm	2.82 ± 0.01 mm	6.0%
